# Mrs. Virginia Asher Dambach

**Published:** 1917-05

**Authors:** 


**Mrs. Virginia Asher Dambach,** widow of Dr. John Dambach of Buffalo, died April 12 at Little Falls, N. Y.

